# Analyzing the effects of physical activity levels on aggressive behavior in college students using a chain-mediated model

**DOI:** 10.1038/s41598-024-55534-3

**Published:** 2024-03-09

**Authors:** Haohan Yu, Qiaochu Ma, Yao Sun, Shan Jiang, Songhan Hu, Xin Wang

**Affiliations:** 1 College of Physical Education, China University of Mining and Technology, Xuzhou, China; 2 College of P.E and Sports, Beijing Normal University, Beijing, China; 3 Department of Sociology, London School of Economics and Political Science, Houghton St, London, WC2A 2AE UK; 4 Department of Sports Science and Physical Education, The Chinese University of Hong Kong, Shatin, Hong Kong; 5 School of Physical Education (Main Campus), Zhengzhou University, Zhengzhou, China

**Keywords:** Physical activity, Self-efficacy, Self-control, Aggressive behaviors, Chain mediating effect, Psychology, Human behaviour

## Abstract

This study aims to examine the mediating role of self-efficacy (SE) and self-control (SC) in the relationship between physical activity (PA) and aggressive behaviors (AB) among college students. It provides a basis for the prevention and control of AB among college students. This study employed a survey research methodology, including the PA Level Scale, the General Self-efficacy Scale, the Self-control Scale, and the Chinese Aggressive Behaviors Scale on 950 college students. The chain mediating effect test and Bootstrap analysis were applied. The results were as follows: (1) There was a main effect of PA on SE, SC, and AB as well as all sub-indicators (physical aggression, verbal aggression, anger, hostility, self-directed aggression), i.e., PA had a direct effect on the control of all three; (2) PA level was significantly negatively correlated with AB and significantly positively correlated with SE and SC. That is, the higher the level of PA, the better the SE and SC, and the lower the probability of AB; (3) The three pathways had mediating effects: PA → SE → AB, PA → SC → AB, PA → SE → SC → AB, with effect sizes of 8.78%, 28.63%, and 19.08%, respectively. It is concluded that regular PA is a potent method for decreasing aggressive behavior and psychological issues in university students while additionally promoting self-efficacy and self-control. Increasing the intensity of PA may enhance the effectiveness of these chain benefits.

## Introduction

Aggressive behaviors (AB) involve intentional physical or psychological harm to others^1^. Such behaviors not only cause serious injury to the victims but also increase the likelihood of future criminal behavior if left unchecked. This poses a significant threat to the attacker and societal stability^2^. AB is a significant issue in schools, often manifesting as bullying or other forms of violence. It is crucial to address this problem effectively to ensure a safe and healthy learning environment for all students. A study involving seven countries found that about 30% of adolescents have experienced bullying^3^. Even in college, instances of physical assault and school bullying persist. According to studies, around 10–15% of undergraduate students in the United States encounter different forms of AB, which can lead to long-lasting psychological issues. In China, the prevalence of campus violence is alarmingly high, with 38.7% of college students being affected^4^. Currently, the problem of campus violence in colleges and universities is serious. Thus, it has been essential to explore the mechanism of college students' AB and seek effective measures to prevent violence in colleges and universities.

Social control theory believes that the necessary restraints on human animal nature through some force limit the occurrence of various unfavorable destructive behaviors^5^. The social control function of sports has been recognized by psychologists, with Fromm, a reputable American psychologist, considering sports as a means of "anesthetizing social defects." Psychologist Janowitz's a vital theory of social control of sports believes that physical activity (PA) plays a vital role in teaching social norms, creating good interpersonal relationships and atmosphere, improving mutual understanding, and directly relieving the psychological stress of social members^6^. At the same time, the World Health Organization (WHO) has demonstrated that PA is a potent tool for enhancing students' physical and psychological health and academic outcomes^7^. Furthermore, regular PA has been proven to reduce AB^8^. In recent years, a study has shown that college students with higher levels of PA were less likely to have AB compared to sedentary people^9^. While a substantial correlation between PA and AB has been shown, the impact of different PA levels on AB enhancement varies. Yuxin's research demonstrates that consistent PA can be effective in decreasing AB in adolescents^10^. Conversely, Khazaie discovered a connection in which increased PA levels were linked to decreased probabilities of AB^11^. So far, the dosing of PA in AB interventions has not been clearly defined.

In addition, self-efficacy (SE) and self-control (SC) are significant mediators in numerous psychological mechanisms studies. They affect mental pathways involved in various behaviors to varying degrees. SE and SC, as important psychological intermediary factors, are important components of an individual self-consciousness and important internal factors of individuals^12^. Bandura's SE theory holds that SE influences SC^13^. Evidence suggests that SE can directly affect self-regulation^14^. The higher the individual's SE, the better his or her SC is. It also indicates that SE plays an important role in constructing an individual's SC. Furthermore, Tongnian discovered a significant chained effect between PA and SE. Specifically, PA was effective in reducing low self-esteem in adolescents, and SE showed a slight increase as a mediator^15^. Zhihao observed a comparable mechanism of action between SC and SE in their ability to enhance PA and reduce internet addiction^16^. As for AB, prior studies have established that both SE and SC significantly predict negative effects on AB^17^. However, more research is needed to fully understand the psychological mechanisms and pathway characterization of enhancing AB in PA.

Thus, this study examines college students as the research subjects and uses PA levels as the primary independent variable to explore the distinctions in AB and the current status attributes of each dimension in different groups. The correlations between PA, SE, SC, and AB were analyzed through mediated chain modeling, examining possible psychological mechanisms. At the same time, based on previous studies and experimental preconceptions, they suggest the following hypotheses: (1) Higher levels of PA are associated with lower levels of AB due to a direct effect between the two. (2) PA positively predicts SE and SC scores, with higher PA levels leading to higher scores. (3) A significant chain-mediated response exists between PA → SE/SC → AB. The aim is to offer theoretical references and data to promote college students' mental health and problem behaviors.

## Method

### Participants

A total of 977 college students were included in this study. The study participants were selected from four universities in Beijing, Shandong, and Inner Mongolia provinces. The final study subjects were randomly sampled from each class. 855 valid questionnaires (19.96 ± 2.68 years) were obtained, with an effective rate of 90%, including 497 males (20.02 ± 3.169) and 358 females (19.66 ± 1.791 years). The study was conducted from October to December 2022 and was approved by the Ethics Committee of the College of Physical Education and Sport in Beijing Normal University (TY20220629), while participants signed an informed consent form. The study was conducted in accordance with the Declaration of Helsinki (1964) and related guidelines and regulations.

The inclusion criteria of the subjects: (1) College students (full-time only), including vocational colleges, and undergraduate colleges; (2) Voluntary participation in the investigation of this study; (3) Physical health, not included in clinical patients.

### Data collection and procedure

This study employed a survey research methodology. The questionnaires are distributed by sojump (a questionnaire collection system developed by Tencent, China). This study employed Harman's single-factor test to calibrate for potential methodological biases associated with this approach^18^. The results showed that 11 factors with eigenvalues greater than 1 were extracted by exploratory factor analysis. And the variance explained by the first factor was 23.599%, which was much lower than the critical value of 40%, which indicated that the data in this study were not affected by the common method bias.

The staff explained the purpose and method of the study to the respondents face to face and solved their doubts in person. With the consent of the counselor (or head teacher) and the person himself or herself, the questionnaire was filled out anonymously, without involving personal information such as student number. The returned questionnaires were strictly screened to exclude invalid responses, short responses (less than 200 s), and 10 consecutive questions with the same options and responses with a high degree of similarity.

Four scales including physical activity rating scale-3 (PARS-3), general self-efficacy scale (GSES), self-control scale (SCS), and Chinese version of Buss&Perry aggression questionnaire (AQ-CV) were selected as the measurement instruments for each variable^19^. Much information about the scales, like their usage, evaluation, acceptance in Chinese studies, and the aspects of the scales examined, were taken into consideration before they were settled. The PARS-3, compiled by Japanese scholar Koyo Hashimoto and translated and revised by Chinese scholar Liang Deqing et al. is a frequently used tool for evaluating college students' PA levels^20^. The GSES developed by Schwarzer in 1993, evaluates individuals' perception of their capacity to handle decision-making situations confidently and competently in psychological experiments^21^. The scale initially included 20 items but was revised to 10 in 1995^22^. The SCS, developed by Tangney and colleagues^23^, is a widely used tool for evaluating an individual's self-control level. Its focus is on the ability to regulate and modify internal emotional responses^24^. Buss and Perry created the AQ-CV to assess the potential aggressiveness of individuals and the likelihood of AB. Many researchers have applied the scale to college campuses have recently found high reliability and validity^25^. The information is shown in Table 1.

**Table 1:** Introduction to the measurement instruments.

Measurement instrument	Compiler	Reviser	Measurement method	Retest reliability & total Cronbach's alpha
Physical Activity Rating Scale (PARS-3)	Koyo Hashimoto	Liang Deqing	The scale consists of 3 dimensions: intensity, duration, and frequency. Each dimension has 1 item, which is scored by Likert 5 points. It follows the formula that "intensity × (time-1) × frequency = total PA score". A score of ≤ 19 indicates a light level of PA, 20 to 42 indicates a medium level of PA, and ≥ 43 indicates a high level of PA	retest reliability: 0.82 total Cronbach's alpha: 0.774
General Self-efficacy Scale (GSES)	Schwarzer	Wang Caikang and Hu Zhongfeng	The scale consists of 10 items, and is scored on a 4-point Likert scale, with scores from 1 to 4 corresponding to "not at all correct" to "completely correct", with higher scores indicating stronger general SE	retest reliability: 0.83 total Cronbach's alpha: 0.97
Self-control Scale (SCS)	Tangney	Tan Shuhua	The scale consists of 5 dimensions: impulse control, health habit, temptation resistance, work concentration, and entertainment moderation. 19 items were selected from the initial 36 items and scored on a 5-point scale, with scores ranging from 1 to 5 corresponding to " completely inconsistent " to " completely consistent ", among which questions 1, 5, 11, and 14 are scored positively, and the remaining items are scored backwards. The higher the total score, the stronger SC	retest reliability: 0.85 impulse control alpha: 0.932 healthy habits alpha0.927 resisting temptation alpha: 0.913 focusing on work alpha: 0.906 abstaining from entertainment alpha: 0.916 total Cronbach's alpha: 0.97
Chinese version of Buss&Perry aggression questionnaire (AQ-CV)	Buss and Perry	Li Xianyun	The scale consists of 5 dimensions: physical aggression, verbal aggression, anger, hostility, and self-directed aggression, including 30 items and is scored on a 5-point Likert scale. The scores range from 1 to 5 corresponding to “inconsistent”, “less consistent”, “half consistent”, “mostly consistent” and “completely consistent”. The higher the total score, the stronger AB	retest reliability: 0.85 physical aggression alpha: 0.968 verbal aggression alpha: 0.953 Anger: 0.911 Hostility: 0.871 self-directed aggression: 0.897 total Cronbach's alpha: 0.980

First, contact the target school and determine the time and place. Then the questionnaires are distributed by sojump. The staff explained the purpose and method of the study to the respondents face to face and solved their doubts in person. With the consent of the counselor (or head teacher) and the person himself or herself, the questionnaire was filled out anonymously, without involving personal information such as student number. The returned questionnaires were strictly screened to exclude invalid responses, short responses (less than 200 s), and 10 consecutive questions with the same options and responses with a high similarity.

### Statistical methods

The four indicators of SE, SC, PA, and AB were tested by normality test using SPSS 21.0 with all P > 0.05 indicating a normal distribution. Differences in total amount between Light/Medium/High levels PA groups were compared and calibrated by one-way ANOVA (ANOVA). Then descriptive statistics, reliability test, and Pearson correlation analysis were carried out. Structural equation modeling (SEM) was developed using Amos 24.0 software to fit the chained mediated effects model and analyze the model fit. Finally, after standardizing each research variable, the macro program process in SPSS was used to test the significance of the mediation effect through the model 6 in the macro program process plug-in in SPSS was used for chain mediated effects testing and confidence interval estimation.

## Results

### Analysis of differences in variables among different levels of physical activity

The main effect of PA levels was statistically significant on AB (F = 24.97, P < 0.01, η^2^ = 0.064) and other dimensions including physical aggression (F = 19.8, P < 0.01, η^2^ = 0.043), verbal aggression (F = 22.08, P < 0.01, η^2^ = 0.055), anger (F = 28.06, P < 0.01, η^2^ = 0.062), hostility (F = 19.59, P < 0.01, η^2^ = 0.045), and self-directed aggression (F = 26.59, P < 0.01, η^2^ = 0.062) i.e., PA had an effect on AB. Post hoc multiple comparisons revealed that on the dimensions of AB, physical aggression, verbal aggression, anger and self-directed aggression, high level of PA was significantly lower than medium and light. In other words, high level of PA compared to medium, and light showed fewer problematic symptoms of AB. At the dimensional level, most of the sub-indicators showed a decreasing trend with PA levels, i.e., the higher the PA level, the lower the scores of each indicator. Among them, except for anger, physical aggression, hostility, and self-directed aggression showed a significant stepwise decrease with PA levels, i.e., high < medium < low, and were significantly different from each other (physical aggression: light-medium: P = 0.0376, η^2^ = 0.063, 95% CI = [0.128, 0.304]; light-high: P < 0.01, η^2^ = 0.079, 95% CI = [0.183, 0.496]; medium–high: P < 0.01, η^2^ = 0.071, 95% CI = [0.273, 0.554]; verbal aggression: light-medium: P = 0.0169, η^2^ = 0.059, 95% CI = [−0.301, −0.153]; light-high: P = 0.001, η^2^ = 0.068, 95% CI = [−0.459, −0.122]; medium–high: P < 0.01, η^2^ = 0.067, 95% CI = [−0.547, −0.263]; anger: light-medium: P = 0.059, η^2^ = 0.089, 95% CI = [−0.128, −0.022]; light-high: P < 0.01, η^2^ = 0.053, 95% CI = [−0.259, −0.050]; medium–high: P < 0.01, η^2^ = 0.076, 95% CI = [−0.558, −0.258]; hostility: light-medium: P = 0.0242, η^2^ = 0.051, 95% CI = [−0.294, −0.073]; light-high: P < 0.01, η^2^ = 0.079, 95% CI = [−0.294, −0.059]; medium–high: P < 0.01, η^2^ = 0.080, 95% CI = [−0.624, −0.327]; self-directed aggression: light-medium: P = 0.0450, η^2^ = 0.088, 95% CI = [−0.191, −0.024]; light-high: P < 0.01, η^2^ = 0.062, 95% CI = [−0.586, −0.239]; medium–high: P < 0.01, η^2^ = 0.075, 95% CI = [−0.506, −0.194]).

In addition, the main effect of PA level was also reflected in SE and SC, i.e., there was a direct effect of PA level on the control of the two (SE: F = 22.69, P < 0.01, η^2^ = 0.05; SC: F = 26.55, P < 0.01, η^2^ = 0.06). The results showed that the relationship between the level of the two and the level of PA was opposite to that of AB, i.e., the higher the level of PA, the higher the level of SE and SC. Specifically: high > medium > light (SE: light-medium: P < 0.01, η^2^ = 0.062, 95% CI = [−0.399, −0.154]; light-high: P < 0.01, η^2^ = 0.059, 95% CI = [−0.548, −0.315]; medium–high: P = 0.004, η^2^ = 0.053, 95% CI = [−0.259, −0.050]; SC: light-medium: P = 0.018, η^2^ = 0.061, 95% CI = [−0.252, −0.086]; light-high: P < 0.01, η^2^ = 0.068, 95% CI = [−0.535, −0.268]; medium–high: P < 0.01, η^2^ = 0.061, 95% CI = [−0.410, −0.169]). This also suggests that increasing weekly PA levels among college students may be an effective means of increasing SE and SC, as shown in Table 2.

**Table 2:** Analysis of differences in variables by physical activity groups (M ± SD).

PA Levels	N	SE	SC	AB	Physical aggression	Verbal aggression	Anger	Hostility	Self-directed aggression
Light	342	2.7 ± 0.65	3.05 ± 0.67	3.7 ± 0.82	3.75 ± 0.89	3.67 ± 0.88	3.92 ± 0.9	3.39 ± 0.97	3.72 ± 0.76
Medium	274	2.93 ± 0.73	3.28 ± 0.85	3.55 ± 0.94	3.49 ± 0.69	3.62 ± 0.67	3.82 ± 0.69	3.15 ± 0.68	3.19 ± 0.52
High	239	3.01 ± 0.66	3.53 ± 0.82	3.17 ± 0.97	3.24 ± 0.7	3.13 ± 0.65	3.31 ± 0.67	2.86 ± 0.67	2.93 ± 0.57
F		22.69**	26.55**	24.97**	19.8**	22.08**	28.06**	19.59**	26.59**
Partial η^2^		0.05	0.06	0.06	0.04	0.05	0.06	0.04	0.06
R^2^		0.05	0.06	0.05	0.04	0.05	0.05	0.04	0.05

*P < 0.05, **P < 0.01.

### Analysis of the relationship among physical activity, self-efficacy, self-control, and aggressive behaviors among college students

**Figure 1:**
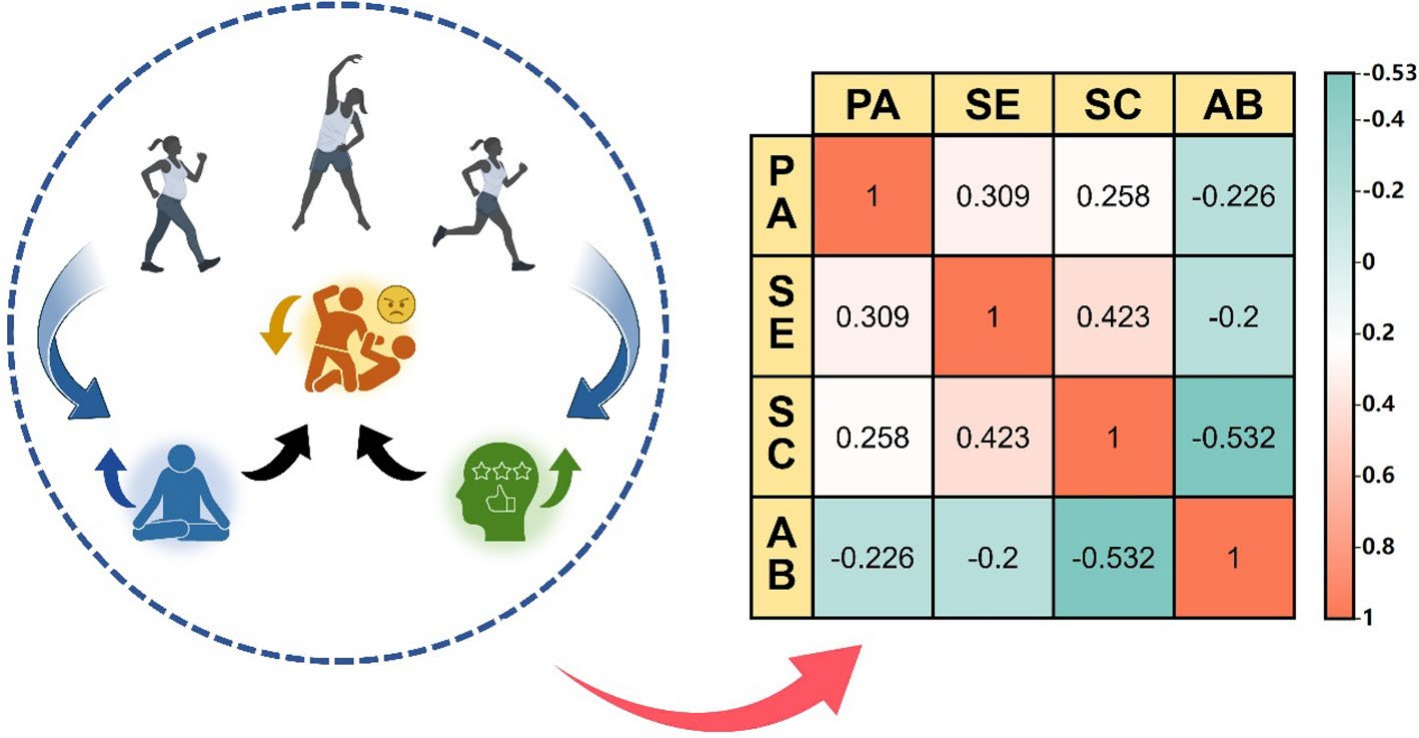
Correlation heatmap between physical activity and individual variables.

The Pearson correlation analysis showed that higher levels of PA were found to be significantly and positively correlated with both SE and SC. This suggests that increased PA leads to higher SE and SC scores (r = 0.309, P < 0.001; r = −0.258, P < 0.001). At the same time, PA levels are inversely related to AB scores, both increasing PA levels decreases AB (r = −0.226, P < 0.001). SE was significantly positively correlated with SC (r = 0.423, P < 0.001) and negatively correlated with AB (r = −0.2, P < 0.001). The significant correlations among the main variables indicated that further tests for mediating effects could be conducted^26^, as shown in Table 3, Fig. 1.

**Table 3:** Descriptive statistics and correlation analysis of each variable.

Variables	M ± SD	PA	SE	SC	AB
PA	3.82 ± 0.80	1.00	–	–	–
SE	2.88 ± 0.70	0.309***	1.00	–	–
SC	3.26 ± 0.80	0.258***	0.423***	1.00	–
AB	3.50 ± 0.93	−0.226***	−0.2***	−0.532***	1.00

*P < 0.05, **P < 0.01, ***P < 0.001.

### Physical activity and aggressive behaviors: a chain mediating effect test

This study uses Amos 24.0 to conduct SEM analysis to examine the mediating effect of SE and SC between PA and AB. The fitting index of the model was: χ2/df = 3.713, P < 0.001, GFI = 0.952, CFI = 0.976, TLI = 0.052, RMSEA = 0.034, 95% CI = 0.056 (0.055–0.058) which met the requirements of psychometrics. The equation model uses the levels of PA as the independent variable with SE and SC as the mediating variable as well as AB as the dependent variable for the mediating effect analysis. The results are showed that PA positively predicted SE (β = 0.254, P < 0.001). PA positively predicted SC (β = 0.041, P < 0.001). PA negatively predicted AB (β = −0.036, P < 0.001). SE positively predicted SC (β = 0.48, P < 0.001). SE negatively predicted AB (β = −0.043, P < 0.001). SC negatively predicted AB (β = −0.551, P < 0.001), as shown in Table 4.

**Table 4:** Regression analysis of chain mediation model of self-efficacy and self-control (n = 855).

Variables	Equation 1: (dependent variable: SE)			Equation 2: (dependent variable: SC)			Equation 3: (dependent variable: AB)		
	β	SE	t	β	SE	t	β	SE	t
PA	0.254	0.032	8.023**	0.15	0.041	3.635**	−0.036	0.022	−5.073**
SE	–			0.49	0.048	10.187**	−0.043	0.026	−3.587**
SC	–			–			−0.551	0.039	−13.089**
R^2^	0.095	–		0.197	–		0.305	–	
F	70.655**	–		135.527**	–		89.268**	–	

**P < 0.01.

Also, the mediation effects of each mediation path were examined, and confidence intervals were estimated by Bootstrap method (5000 repetitions of sampling) The results showed that the Bootstrap 95% confidence intervals for three indirect effect paths of SE and SC as mediating variables did not contain 0, including PA → SE → AB, PA → SC → AB, and PA → SE → SC → AB. The results indicated that the effects of these pathways were significant, as shown in Table 5.

**Table 5:** Bootstrap analysis of the mediating effect test.

Type of effect	Pathway	Effect value	BootSE	Boot95% CI		Effect value ratio (%)
				Lower limit	Upper limit	
Direct effect	PA → AB	−0.114	0.036	−0.185	−0.042	40.28
Indirect effect	PA → SE → AB	−0.053	0.023	−0.097	−0.026	18.72
	PA → SC → AB	−0.085	0.018	−0.127	−0.042	30.04
	PA → SE → SC → AB	−0.031	0.009	−0.095	−0.023	10.95
Total indirect effect	–	−0.169	0.018	−0.187	−0.131	59.72
Total effect	–	−0.283	0.039	−0.319	−0.167	100

**Figure 2:**
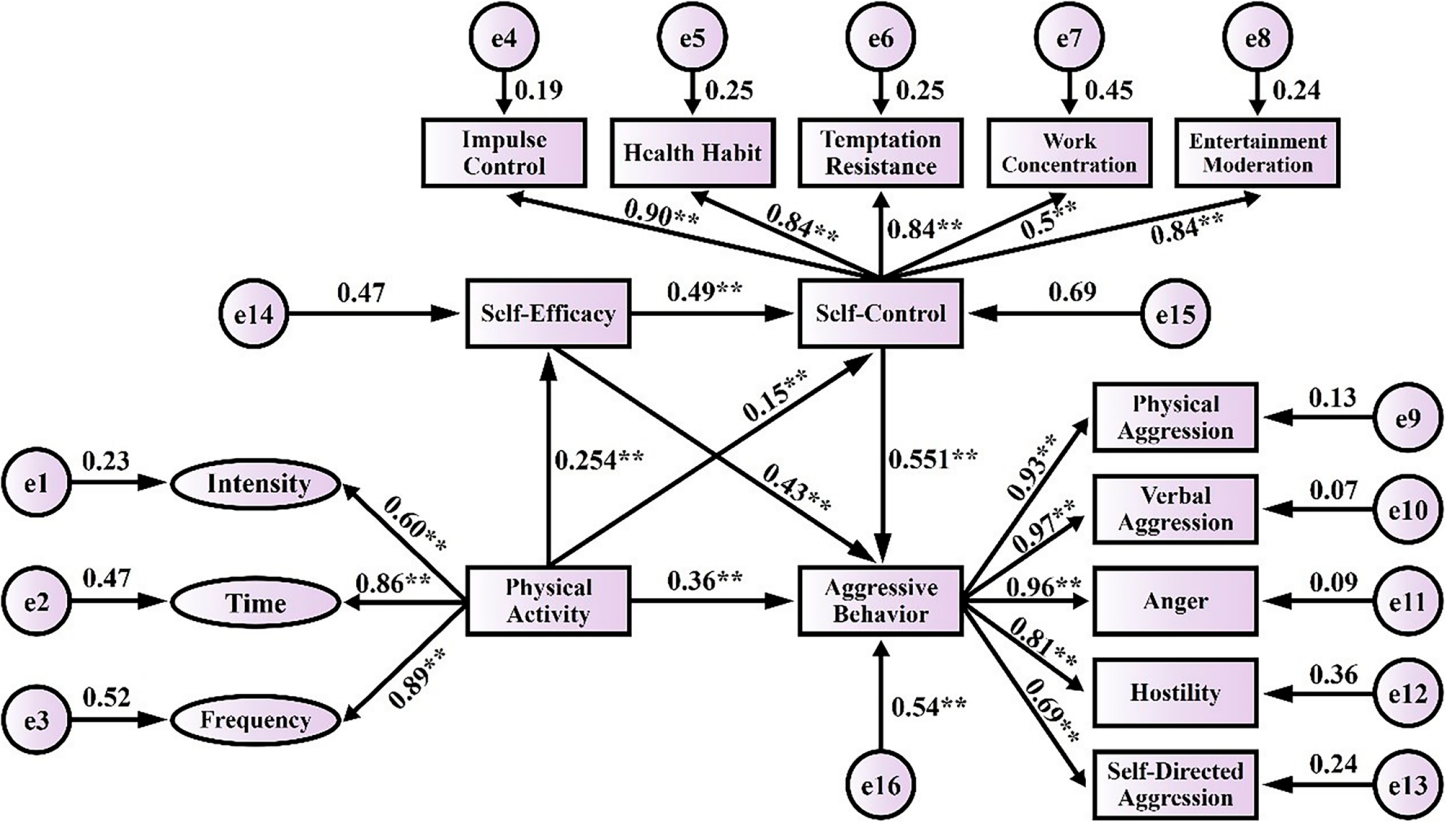
Chain mediation model of self-efficacy and self-control between physical activity and aggressive behaviors.

The mediating effect of SE and SC in the effect of PA on AB among college students was significant. The mediating effect consisted of the indirect effects from the three mediating paths, where the total standardized indirect effect value was −0.148, accounting for 59.72% of the total effect. The results of the indirect effects of the three mediating pathways were as follows: (1)"PA → SE → AB" The effect value of the pathway was −0.023 accounting for 18.72% of the total effect value. (2) "PA → SC → AB" The effect value of the pathway was −0.075, accounting for 30.04% of the total effect value. (3) "PA → SE → SC → AB" The effect value of the pathway was −0.05, accounting for 10.95% of the total effect value. In addition, the effect value of PA and AB was −0.114, accounting for 40.28% of the total effect value. The SEM is shown in Fig. 2.

## Discussion

The occurrence of AB is prevalent among adolescent populations. However, cross-cutting studies such as sports psychology have identified the existence of AB in college student populations^27^. Multiple factors and psychological mechanisms contribute to the occurrence of AB, including innate genetic and neurotransmitter transmission characteristics, as well as acquired factors such as family environment, socio-cultural influences, and cognitive disorders. Timely detection and intervention are essential as failure to do so may result in the development of severe behaviors, such as bullying or even suicide. In recent years, research has demonstrated that PA can be an effective intervention to improve AB, in addition to psychological counseling and pharmacological interventions^10^. The intervention process can also positively promote psychological states such as SE and SC^16^. Based on the investigation, this study found a significant direct improvement effect of PA on AB. Furthermore, SE and SC were also improved accordingly in the pathway of PA → SE/SC → AB. Consequently, the effect of PA on AB may surpass other means, such as psychological counseling or drugs, and provide a novel viewpoint for AB intervention and treatment.

In this cross-sectional survey, it was found that the level of PA was directly related to college students' AB factors and was significantly negatively correlated. The study results showed that the higher the weekly PA level of college students, the lower the expression level of their AB factors, including physical aggression, verbal aggression, anger, hostility, and self-directed aggression. Therefore, hypothesis (I) was fully accepted. This outcome implies that increasing PA participation could effectively control AB and minimize negative outcomes among college students. Additionally, this study revealed that PA has a direct negative correlation with college students' alcohol consumption behavior, as supported by previous research. Nebojša discovered that following a 4-week post-school volleyball program with adolescents, which corresponds to moderate to high-level PA, all participants experienced considerable reductions in AB levels, alongside further enhancements in physical fitness and athletic performance^28^.Not only did Qi find, through a study of 1,000 college students, that those with higher levels of PA demonstrated lower AB scores, but they also experienced a greater sense of well-being^29^. Similar to this study, Yuxin discovered that PA programs involving groups had greater effect sizes than stand-alone programs in improving adolescent AB. This implies that increasing PA levels may not be the sole method to enhance the effectiveness of AB interventions^10^.

The effectiveness of PA in improving cognitive control can be explained by several psychological mechanisms. Baumeister's Ego Depletion Theory proposes that individuals have a limited willpower resource which can be enhanced by PA. Regular PA can improve performance on sustained tasks requiring SC and decrease the occurrence of AB^30^. Jason and Mark's Strategic Resource Depletion Theory proposes that the continuous performance of SC tasks depletes psychological resources. College students with higher levels of PA are more likely to experience a greater depletion of psychological resources that support self-regulation and, consequently, exhibit a reduction in problematic behavior, including AB^31^. These findings all support the theory of AB from the social learning perspective. Besides, they provide strategic and theoretical support for intervention and control programs for AB among college students. Engaged in PA, students not only can prevent and control AB by changing their social skills, releasing excess energy, improving their mental health, and regulating emotions^32^, but also, in this way, a portion of aggression can also be released. Therefore, most of the adolescents who usually maintain regular PA have a good level of physical and mental health and more standardized behaviors^33^.

Based on the survey's SE results, the study's second finding concludes that SE partially mediates PA's effect on college students' AB. SE's chain type increases with the PA level, and its mediating effect accounts for 8.78% of the total effect value. Although no significant differences in SE were observed between those with high and moderate levels of PA, the above results still suggest that the hypothesis (II) that "PA positively predicts SE and SC scores" is accepted. However, increasing the level of PA remains an effective strategy for reducing college students with elevated stress levels compared to those with low PA levels. It also suggested that PA could indirectly reduce or mitigate college students’ AB by enhancing SE. The research devoted to SE and AB in the past is severely limited, with most studies being conducted in specific application scenarios or for experimental purposes. For instance, Chen investigated SE's function as a mediator, positively predicting emotion regulation^34^. Cao found that PA had a significant facilitating effect on college students' academic-related SE^35^. However, like the present study, recent studies have also confirmed the potential correlation between SE and AB. Worthy and Magalhães similarly discovered a significant correlation and negative predictive relationship between SE and AB in constructing a path-structural model of the relationship between psychological harm and life satisfaction^36,37^. Subsequently, Rottweiler's thorough analysis of SE factors found that disparities in SE levels mediate differences in individual cognition, emotionality, and behavior. Additionally, high SE levels display a strong relationship with positive self-worth, academic proficiency, and overall quality of life for university students^38^. Effective regulation of SE may positively contribute to college students' mental health development, the establishment of proper values, and the prevention and control of undesirable behaviors.

Except for SE, the third finding was that SC played a partially mediating role in the effect of PA on AB among college students, with its mediating effect accounting for 28.63% of the total effect value. Therefore, the conjecture in hypothesis (III) for the two paths PA → SE/SC → AB is also well accepted. Consistent with previous studies, the present study supports Zhang's findings that SC serves as a buffer and protective factor against PA and college student AB^39^. Additionally, the current findings support Pesce's perspective that PA can indirectly mitigate and regulate AB of college students through the promotion of SC^40^. Baumeister notes that SC can be strengthened through regular PA, much like a muscle. It has also been found that this ability can transfer to other areas of life^41^. And several psycho-theoretical models can explain this negative predictive effect of SC to AB. SC in a certain field can be improved through training, and such ability can be spread to other fields, which brings light to improving teenagers' SC ability to reduce their AB. Based on the dual-system theory of SC^42^, SC is composed of two systems of impulse and control (also known as rationality and emotion). The impulse system is only the automatic emotional response and behavioral association trend of the human body to stimulation, which does not require individual cognitive participation. The control system, on the other hand, with superior characteristics, inhibits impulsive responses and is responsible for emotion management and decision-making^43^. Individuals with high control system scores develop higher-order evaluation and suppression criteria when faced with impulsivity due to higher rational psychological literacy and greater consideration of future consequences^44^. Individuals with high SC can regulate their emotions and behaviors to achieve their goals, rationally control their behaviors, and avoid the development of AB symptoms. As an important personal quality of the will, SC is closely related to AB. Individuals with greater SC had lower rates of AB. Therefore, PA, as an important means to improve the SC ability, can effectively prevent or alleviate the AB of college students.

To sum up, most previous studies explored the psychological mechanism of college students' AB from the perspective of SC. This study innovatively found the relationship between SE, PA, and AB. On this basis, it further found that SE and SC in PA and the chain mediation between college students’ aggression. It was found that SE and SC play a significant mediating role in the influence of PA on college students’ AB, with an effect value of 19.08% of the total indirect effect. This supports the idea of self-regulation and ternary interactive determinism of social cognition theory, i.e., the interactive process of individual internal factors, behaviors, and environment^45,46^. On the one hand, it proves the influence and regulatory effects of SE and SC on individual AB. On the other hand, it also proves that SE and SC play a mediating role in the PA and AB chains. Under the influence of PA, if SE and SC, which are promoted by PA, are strong, it will influence the individual internal factors to further inhibit the AB of college students. Therefore, the more PA college students participate in, the greater their SE and SC will be. So, regular PA may gradually contribute to the formation of a psychologically virtuous circle, which in turn influences the improvement of ABal symptoms. Thus, the SE and SC of college students play an important role in PA and AB.

### Implications

This study not only verified the chain-mediated effect of SE and SC in PA and AB, but also extended the theory of prevention and control of aggression, as well as the inhibition of the path of PA, which has some theoretical and practical significance for the prevention and control of aggression.

In terms of theoretical development, although AB, SE, and SC have received significant attention within the mental health field, most research has focused on individual or paired indicators. Conversely, the number of studies that have conducted combination and mediator variable analyses based on the three constructs has been limited, and there is a lack of evidence on PA as a primary variable with interventions. This study is pioneering in incorporating PA as a primary variable and enhancing the dosing characteristics of PA levels in improving AB. In addition, SE and SC were included as mediating variables to examine further reticular advantages in the PA improvement pathway in AB. We provide data references that augment theories of sport psychology and cross-psychology.

In recent years, many psychology research centers have focused on the mechanism of AB formation, lacking effective intervention treatments. To address individual factors of AB, cultivating good behavioral habits such as regular PA can bring the interlocking mediating effect of the two into full play. In this study, we found that we can effectively reduce the likelihood of AB by cultivating good behavioral habits of regular PA to address the individual factors of AB. Meanwhile, increasing the intensity of weekly PA can strengthen the mediating chain effect of SE and SC in the pathway of "PA improves AB", and activate one's own motivation to actively inhibit the uncontrolled impulses and problem behaviors of individuals. Therefore, the present study provides practical exercise prescription and activity guidelines for reducing AB and maintaining the mental health of college students on college campuses in the future.

### Limitations and suggestions for future research

The study aimed to offer a psychologically-based theoretical model and PA-specific guidelines to improve AB among college students. The investigation explored the effectiveness and dosage characteristics of PA in AB interventions. However, the study presents several limitations and shortcomings in three aspects.

In this study, the correlation analysis was carried out for each of the four indicators and the final data were combined. However, the analysis was not refined according to the PA level, which may result in errors and heterogeneity in the correlation results between the variables. Therefore, the study may lack scientific basis and rigor.

There are various methods to define PA within the athletic training field. However, differences in PA levels are inadequate to depict the complete PA dose in AB interventions. For instance, in PA, the physiological and psychological indicators of individuals can be affected by different factors, including the form of PA, program characteristics, scenario, or exercise experience. Using only PA level as the independent variable may result in a lack of rigor in the dose characteristic outcomes. Additionally, recommendations in this study were limited to college students and were based on the results influenced by various PA levels. However, specific guidance programs and implementation methods were not included in the recommendations.

Therefore, in future studies, it would be beneficial to present differences in PA by incorporating various factors such as PA form, intensity, and program differences. Additionally, incorporating indicators such as self-esteem, positive thinking, and mental toughness in the mediator chain model could enrich the SEM and mental path mechanism based on PA intervention AB. It offers a dependable and pragmatic PA plan as well as a theoretical foundation to enhance mental health and address behavioral difficulties among college students.

## Conclusion

(1) PA is an effective method for reducing aggressive behaviors and psychological issues amongst college students. Elevating weekly PA levels may enhance the efficiency of this effect; (2) PA level positively predicts self-efficacy and self-control, and increasing PA enhances the benefits of both; (3) Self-efficacy and self-control are crucial components in curbing aggressive behaviors. Improving both can effectively reduce aggressive behaviors.

### Supplementary Information

Supplementary Information.

## Data Availability

The original contributions presented in this study are included in the article/supplementary material, further inquiries can be directed to the corresponding author/s.
