# Supplementary material for: Analyzing the effects of physical activity levels on aggressive behavior in college students using a chain-mediated model
Source: Sci Rep. 2024 Mar 9;14:5795. doi: 10.1038/s41598-024-55534-3 (PMC10924895; doi:10.1038/s41598-024-55534-3)
Supplement: Supplementary file 1 — Supplementary Information. [file 41598_2024_55534_MOESM1_ESM.pdf]

Appendix 1: Physical Activity Rating Scale (PARS-3)

1. How is the intensity of your physical exercise?

A. light exercise (such as walking, doing radio gymnastics, playing gateball, etc.)

B. small intensity less intense exercise (such as recreational volleyball, table tennis, jogging, tai chi, etc.); medium intensity more intense and lasting exercise (such as cycling, running, playing table tennis, etc.)

C. shortness of breath, sweating a lot of large intensity, but not lasting sports (such as playing badminton, basketball, tennis, soccer, etc.)

D. breathing fast, sweating a lot of high-intensity and lasting sports (such as running, sets of aerobics exercises, swimming, etc.)

2. How many minutes do you spend at a time in the above-mentioned intense physical activities?

A. 10 minutes or less

B. 11 to 20 minutes

C. 21 to 30 minutes

D. 31 to 59 minutes

E. 60 minutes or more

3. How many times a month do you do the above-mentioned physical activities?

A. Less than once a month

B. 3 to 5 times a week

C. 2 to 3 times a month

D. About once a day

E. 1 to 2 times a week

4. What kind of physical exercise do you like?

A. Walking, running

B. Travel, excursions

C. Sports dance

D. Ball games

E. Jumping rope

F. Taijiquan, health care

G. Exerciser activity

H. Swimming

I. Other

Appendix 2: General Self-Efficacy Scale (GSES)

1. If you try hard, you can always accomplish a task efficiently.

(1) Totally wrong

(2) Basically right

(3) Almost right

(4) Absolutely right

2 Even objected by others, you still can manage to get what you want.
(1) Totally wrong
(2) Basically right
(3) Almost right
(4) Absolutely right

3. Sticking to your dream and realizing it is easy with no difficulty to you.
(1) Totally wrong
(2) Basically right
(3) Almost right
(4) Absolutely right

4. You have confidence to efficiently response to sudden evens.
(1) Totally wrong
(2) Basically right
(3) Almost right
(4) Absolutely right

5. You maintain that you have the talent to overcome obstacles.
(1) Totally wrong
(2) Basically right
(3) Almost right
(4) Absolutely right

6. If you make the effort, you can solve most of the obstacles.
(1) Totally wrong
(2) Basically right
(3) Almost right
(4) Absolutely right

7. Since you believe that you have the capacity to solve problems, you can keep a cool head when you are confronted with difficulties.
(1) Totally wrong
(2) Basically right
(3) Almost right
(4) Absolutely right

8. When come across a problem, you can always find out several solutions.
(1) Totally wrong
(2) Basically right
(3) Almost right
(4) Absolutely right

9. When come across difficulties, you can always figure out solutions.

(1) Totally wrong
(2) Basically right
(3) Almost right
(4) Absolutely right

10. No matter what happened, you can always easily response to various situations.
(1) Totally wrong
(2) Basically right
(3) Almost right
(4) Absolutely right

Appendix 3: Self-Control Scale (SCS)
1. I am good at resisting temptation
(1) Strongly disagree
(2) Disagree
(3) Agree
(4) Strongly agree

2. I have a hard time breaking bad habits.
(1) Strongly disagree
(2) Disagree
(3) Agree
(4) Strongly agree

3. I am lazy.
(1) Strongly disagree
(2) Disagree
(3) Agree
(4) Strongly agree

4. I say inappropriate things.
(1) Strongly disagree
(2) Disagree
(3) Agree
(4) Strongly agree

5. I do certain things that are bad for me, if they are fun.
(1) Strongly disagree
(2) Disagree
(3) Agree
(4) Strongly agree

6. I refuse things that are bad for me.
(1) Strongly disagree

(2) Disagree
(3) Agree
(4) Strongly agree

7. I wish I had more self-discipline.
(1) Strongly disagree
(2) Disagree
(3) Agree
(4) Strongly agree

8. People would say that I have iron self-discipline.
(1) Strongly disagree
(2) Disagree
(3) Agree
(4) Strongly agree

9. Pleasure and fun sometimes keep me from getting work done.
(1) Strongly disagree
(2) Disagree
(3) Agree
(4) Strongly agree

10. I have trouble concentrating.
(1) Strongly disagree
(2) Disagree
(3) Agree
(4) Strongly agree

11. I am able to work effectively toward long-term goals.
(1) Strongly disagree
(2) Disagree
(3) Agree
(4) Strongly agree

12. Sometimes I can't stop myself from doing something, even if I know it is wrong.
(1) Strongly disagree
(2) Disagree
(3) Agree
(4) Strongly agree

13. I often act without thinking through all the alternatives.
(1) Strongly disagree
(2) Disagree
(3) Agree

(4) Strongly agree

Appendix 4: Aggression Questionnaire (AQ)

1. Once in a while I can’t control the urge to strike another person.
(1) Strongly disagree
(2) Disagree
(3) Agree
(4) Strongly agree

2. Given enough provocation, I may hit another person.
(1) Strongly disagree
(2) Disagree
(3) Agree
(4) Strongly agree

3. If somebody hits me, I hit back.
(1) Strongly disagree
(2) Disagree
(3) Agree
(4) Strongly agree

4. I get into fights a little more than the average person.
(1) Strongly disagree
(2) Disagree
(3) Agree
(4) Strongly agree

5. If I have to resort to violence to protect my rights, I will.
(1) Strongly disagree
(2) Disagree
(3) Agree
(4) Strongly agree

6. There are people who pushed me so far that we came to blows.
(1) Strongly disagree
(2) Disagree
(3) Agree
(4) Strongly agree

7. I can think of no good reason for ever hitting a person.
(1) Strongly disagree
(2) Disagree
(3) Agree
(4) Strongly agree

8. I have threatened people I know.
(1) Strongly disagree
(2) Disagree
(3) Agree
(4) Strongly agree

9. I have become so mad that I have broken things.
(1) Strongly disagree
(2) Disagree
(3) Agree
(4) Strongly agree

10. I tell my friends openly when I disagree with them.
(1) Strongly disagree
(2) Disagree
(3) Agree
(4) Strongly agree

11. I often find myself disagreeing with people.
(1) Strongly disagree
(2) Disagree
(3) Agree
(4) Strongly agree

12. When people annoy me, I may tell them what I think of them.
(1) Strongly disagree
(2) Disagree
(3) Agree
(4) Strongly agree

13. I can't help getting into arguments when people disagree with me.
(1) Strongly disagree
(2) Disagree
(3) Agree
(4) Strongly agree

14. My friends say that I'm somewhat argumentative.
(1) Strongly disagree
(2) Disagree
(3) Agree
(4) Strongly agree

15. I flare up quickly but get over it quickly.

(1) Strongly disagree
(2) Disagree
(3) Agree
(4) Strongly agree

16. When frustrated, I let my irritation show.
(1) Strongly disagree
(2) Disagree
(3) Agree
(4) Strongly agree

17. I sometimes feel like a powder keg ready to explode.
(1) Strongly disagree
(2) Disagree
(3) Agree
(4) Strongly agree

18. I am an even-tempered person.
(1) Strongly disagree
(2) Disagree
(3) Agree
(4) Strongly agree

19. Some of my friends think I'm a hothead.
(1) Strongly disagree
(2) Disagree
(3) Agree
(4) Strongly agree

20. Sometimes I fly off the handle for no good reason.
(1) Strongly disagree
(2) Disagree
(3) Agree
(4) Strongly agree

21. I have trouble controlling my temper.
(1) Strongly disagree
(2) Disagree
(3) Agree
(4) Strongly agree

22. I am sometimes eaten up with jealousy.
(1) Strongly disagree
(2) Disagree

(3) Agree
(4) Strongly agree

23. At times I feel I have gotten a raw deal out of life.
(1) Strongly disagree
(2) Disagree
(3) Agree
(4) Strongly agree

24. Other people always seem to get the breaks.
(1) Strongly disagree
(2) Disagree
(3) Agree
(4) Strongly agree

25. I wonder why sometimes I feel so bitter about things.
(1) Strongly disagree
(2) Disagree
(3) Agree
(4) Strongly agree

26. I know that "friends" talk about me behind my back.
(1) Strongly disagree
(2) Disagree
(3) Agree
(4) Strongly agree

27. I am suspicious of overly friendly strangers.
(1) Strongly disagree
(2) Disagree
(3) Agree
(4) Strongly agree

28. I sometimes feel that people are laughing at me behind my back.
(1) Strongly disagree
(2) Disagree
(3) Agree
(4) Strongly agree

29. When people are especially nice, I wonder what they want.
(1) Strongly disagree
(2) Disagree
(3) Agree
(4) Strongly agree
